# Efficacy of Internet-based cognitive behavioral therapy for reducing perfectionistic strivings in the Republic of Korea: A randomized controlled trial

**DOI:** 10.1016/j.invent.2025.100851

**Published:** 2025-06-29

**Authors:** Sanghoon Oh, Jeong hee Cha, Jungwon Joo, Ji Hyung Lee, Yunna Lee, Hyung Jun Lee, Dong Uk Yoon, Jeonghwan Lee

**Affiliations:** a Department of Psychiatry, Uijeongbu Eulji Medical Center, Eulji University School of Medicine, Uijeongbu, Republic of Korea; b YD Clinic Research Institute, Busan, Republic of Korea; c Department of Psychiatry, Chungbuk National University Hospital, Cheongju, Republic of Korea; d Department of Psychiatry, Kosin University Gospel Hospital, Busan, Republic of Korea; e Department of Psychiatry, Yongin Mental Hospital, Yongin-si, Gyeonggi-do, Republic of Korea

**Keywords:** Internet interventions, Internet-based cognitive-behavioral therapy, Perfectionism

## Abstract

**Background:**

Perfectionistic strivings characterized by excessively high standards, fears of mistakes, and critical self-evaluations can lead to avoidance, worry, procrastination, and self-criticism, negatively impacting mental health. Since individuals with perfectionism are less likely to seek face-to-face therapy, internet-based cognitive behavioral therapy (ICBT) may improve accessibility.

**Objectives:**

This study evaluated the effectiveness of an ICBT program specifically designed to reduce perfectionistic strivings.

**Methods:**

A total of 101 participants with significant perfectionism were randomly assigned to a 5-week unguided ICBT program or a waiting list control group. Online assessments were conducted at baseline and post-intervention using the Frost Multidimensional Perfectionism Scale (FMPS), Patient Health Questionnaire-9 (PHQ-9), Generalized Anxiety Disorder-7 (GAD-7), Perceived Stress Scale (PSS), and Satisfaction with Life Scale (SWLS). Intention-to-treat and completer analyses were performed.

**Results:**

Of 101 participants, 62 (61.4 %) completed both assessments. The ICBT group completed an average of 3.71 out of 5 modules, with 58.8 % completing all. Compared to the control group, the ICBT group showed significant reductions in perfectionistic strivings (FMPS Concern over Mistakes: *d* = −0.65, 95 % CI [−1.05, −0.25]), anxiety (GAD-7: *d* = −0.42, 95 % CI [−0.83, −0.01]), and increased life satisfaction (SWLS: *d* = 0.62, 95 % CI [0.20, 1.03]).

**Conclusions:**

The ICBT program effectively reduced perfectionistic strivings and related symptoms, highlighting its potential as a scalable and accessible intervention. Further studies are warranted to directly compare ICBT with traditional face-to-face CBT and assess the durability of treatment effects.

## Highlights

• Internet-based CBT (ICBT) effectively reduced perfectionistic strivings and associated distress.
• The study is the first RCT of ICBT for perfectionism conducted in South Korea.
• Participants in the ICBT group showed significant improvements in anxiety and life satisfaction.
• An unguided ICBT program demonstrated efficacy comparable to guided interventions.
• Findings highlight ICBT's potential as a scalable, accessible treatment for perfectionism.

## 1 Introduction

Perfectionism is a multidimensional construct characterized by a combination of cognitive and behavioral patterns (Frost et al., 1990). Although earlier literature distinguished between adaptive and maladaptive forms of perfectionism, recent theoretical discussions highlight significant problems with this dichotomy, arguing that no form of perfectionism is genuinely adaptive (Gaudreau, 2019; Greenspon, 2000). Instead, contemporary frameworks differentiate rigid, unhealthy ‘perfectionistic strivings’—characterized by an inflexible pursuit of flawlessness and severe self-criticism—from ‘excellencism,’ a flexible, realistic strivings toward high standards and continual improvement (Gaudreau et al., 2022; Madigan et al., 2018). Such perfectionistic strivings correspond to what is often labeled ‘clinical perfectionism,’ characterized by unrealistically high standards and chronic self-evaluation against those standards (Limburg et al., 2017). These perfectionistic tendencies are prevalent across both clinical and non-clinical populations (Dahlenburg et al., 2019; Enns and Cox, 1999; Wu et al., 2017) and are associated with significant psychological distress and functional impairment (Egan et al., 2011). They have been linked to a range of psychiatric conditions, including eating disorders, obsessive-compulsive disorder (OCD), anxiety disorders, and depression, and they often contribute to the maintenance of these conditions (Shafran and Mansell, 2001; Shahar et al., 2004; Sirois and Molnar, 2016). Therefore, targeted interventions to reduce rigid and unrealistic perfectionistic strivings are crucial for mitigating the negative impact of perfectionism on mental health.

Cognitive behavioral therapy (CBT) is an evidence-based psychological intervention premised on the understanding that maladaptive thoughts, emotions, and behaviors interact and reinforce each other, contributing to psychological distress (Beck et al., 2024; Butler et al., 2006). CBT seeks to alleviate this distress by systematically identifying, challenging, and modifying distorted cognitions and maladaptive behavioral patterns through structured techniques, such as cognitive restructuring, behavioral experiments, and exposure exercises (Beck, 2020). CBT has been identified as a critical intervention for addressing clinical perfectionism (Mahmoodi et al., 2020; Steele et al., 2013), grounded in its empirical approach to modifying maladaptive behaviors and cognitions. A meta-analysis of 15 randomized controlled trials on CBT for perfectionism demonstrated the efficacy of CBT in reducing perfectionism symptoms (Galloway et al., 2022). Whether administered through traditional face-to-face sessions or self-help approaches, CBT significantly decreased perfectionism and concurrently alleviated associated conditions such as depression, anxiety, and eating disorders, compared to control groups (Galloway et al., 2022). Although traditional CBT is effective, significant logistical and accessibility-related challenges often hinder its widespread application (Andersson et al., 2014; Carrier et al., 2022). These challenges include the limited availability of qualified therapists, geographic barriers for those living in remote or rural areas, scheduling constraints due to employment or academic responsibilities, and stigma associated with attending mental health services in person (Bekker et al., 2017; Carrier et al., 2022). To overcome these barriers, it is recommended to provide evidence-based treatments through easily accessible methods such as Internet-based interventions.

Internet-Based Cognitive Behavioral Therapy (ICBT) emerges as a cost-effective alternative (Nordgren et al., 2014), offering necessary adaptability for implementation in real-world settings where symptoms manifest. It has been shown to be effective for a wide range of psychiatric and psychological conditions in numerous controlled trials (Andersson, 2016). Notably, a study investigating ICBT for clinical perfectionism reported moderate to large effect sizes for the treatment on two primary outcome measures: the Concern over Mistakes and Personal Standards subscales of the Frost Multidimensional Perfectionism Scale (FMPS) (Frost et al., 1990; Rozental et al., 2017). A recent randomized controlled trial (RCT) comparing the efficacy of ICBT with traditional face-to-face CBT found no significant differences (Shu et al., 2019), suggesting the potential of ICBT as an equally effective treatment option for perfectionistic strivings. Nonetheless, research on ICBT specifically targeting perfectionism remains relatively limited compared to the more extensive literature on Internet interventions addressing established psychiatric disorders.

Perfectionistic strivings are a particularly pertinent issue in Korean society, driven by cultural factors such as the intense competition associated with the Korean entrance exam system, high educational aspirations, and the pervasive influence of meritocratic values (Kang et al., 2020; Shin et al., 2023). The culture of high-stakes examinations in Korea, often viewed as a determinant of one's future success, fosters an environment where individuals are expected to achieve exceptional academic outcomes (Kwon et al., 2017). This societal pressure has been linked to perfectionistic tendencies, as students and even adults strive to meet unrealistically high expectations. Moreover, the emphasis on meritocracy and competition exacerbates these tendencies, with perfectionistic strivings manifesting as a significant source of psychological distress among individuals (Curran and Hill, 2019; Park and Jeong, 2015). Addressing perfectionistic strivings in this context is crucial, as it represents an important social and mental health issue in Korea.

This study is the first RCT conducted in Asia to evaluate the efficacy of an unguided ICBT program for individuals experiencing elevated levels of perfectionism. Building on the significance of perfectionism as a multidimensional construct and its heightened relevance in the sociocultural context of Korea, our primary objective was to examine the efficacy and acceptability of the ICBT program in reducing perfectionistic strivings. Additionally, we aimed to investigate its impact on associated psychological states including depression, anxiety, stress, and life satisfaction. We hypothesized that individuals with elevated levels of perfectionism who received ICBT would exhibit significantly lower levels of perfectionistic strivings, related psychological distress, and improved life satisfaction compared to those in the waiting list control group at post-intervention.

## 2 Methods

### 2.1 Participants and procedure

Participants were recruited through advertisements on social media, university campuses, and psychiatric outpatient clinics. The inclusion criteria were: 1) ages between 19 and 65, and 2) a score of 25 or above on the FMPS Concern over Mistakes, and 3) provision of informed consent. Individuals were excluded if they declined to participate or withdrew consent after the screening procedure or study explanation. The cutoff score of ≥25 on the FMPS Concern over Mistakes was selected to target individuals experiencing elevated levels of perfectionism. This cutoff was determined by the combined mean + 1 standard deviation (SD), based on the average and SD of the FMPS Concern over Mistakes from a non-clinical control group of 635 individuals from prior studies (Egan et al., 2011). This study received approval from the Institutional Review Board of Chungbuk National University Hospital (IRB No. CBNUH 2022-06-031), and was retrospectively registered on the WHO International Clinical Trial Registry Platform (Registration No: KCT0010217). Informed consent was obtained from all participants prior to their participation.

**Fig. 1:**
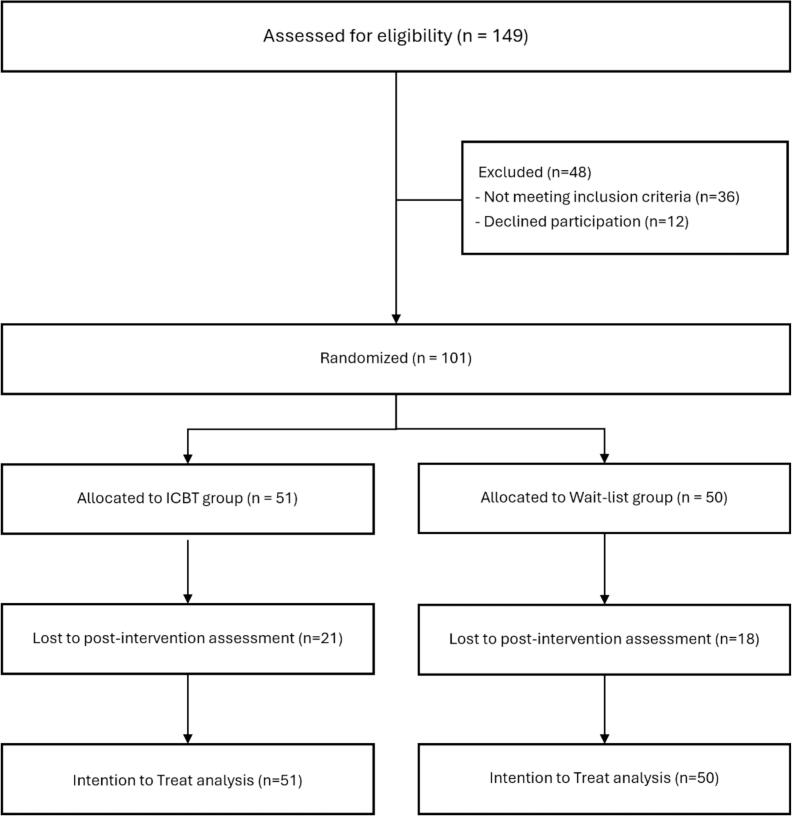
Flow chart of participants detailing recruitment, randomization, and attrition throughout the study.

Of the 149 individuals who expressed interest in the study, 115 met the inclusion criteria. Following a comprehensive study briefing, 101 participants provided written informed consent and were randomized into a parallel-group trial design with a 1:1 allocation ratio. Of these, 75 were recruited through clinic referrals, and 26 were recruited from non-clinic sources, including social media and online bulletin boards. The 51 participants in the ICBT group received a link to begin the self-guided ICBT program and then underwent baseline assessments. Participants who did not start or those who paused the program for more than a week were encouraged by the research team through messages or phone calls to complete the program. A post-intervention assessment was conducted following the completion of the ICBT program. The 50 participants in the waitlist control group underwent baseline assessments, waited for five weeks, completed post-assessments, and then received the link to begin the ICBT program. Both baseline and post-intervention assessments were administered online via secure survey links sent by the research team. Participants completed these assessments on their personal devices at their convenience. A flow chart of participants is shown in Fig. 1.

The average age of participants was 31.55 ± 7.83 years, with the majority being female (71.3 %). For the ICBT group, the mean age was 30.84 years (SD = 7.40), while the waitlist group had a mean age of 31.40 years (SD = 7.56), with no significant age difference observed (*t* = 0.37, *p* = .709). Regarding gender distribution, the ICBT group consisted of 38 females and 13 males, whereas the waitlist group included 34 females and 16 males. Statistical analysis using the chi- square test confirmed that these proportions were not significantly different (*χ*^*2*^ = 0.25, *p* = .615), indicating comparable gender representation across both groups.

### 2.2 Intervention

The self-guided ICBT program developed and utilized in this study consisted of five modules, based on text and video lectures, designed to enable participants to identify and modify maladaptive thoughts (Table 1). Each module took approximately 30 min to complete, with one module recommended per week. However, participants were allowed to review and practice freely, proceeding to subsequent modules at their discretion.

**Table 1:** Contents of the internet-based cognitive-behavior therapy (ICBT) intervention for individuals with elevated levels of perfectionism.

Module	Content
(1) Recognizing perfectionism	Online psychological assessment (pre-intervention) Psychological education through video content Setting therapeutic goals: managing perfectionism to promote well-being Strengthening motivation for change Interactive elements where individuals reflect on and share their personal experiences in response to questions
(2) Adjusting unrealistic standards	Psychoeducation, identifying ideal standards, and shifting to realistic standards Practicing through case examples Utilizing the community feature for peer support
(3) Changing automatic thoughts	Psychological education through video content Identifying and correcting cognitive distortions (cognitive restructuring) Exploring automatic thoughts influencing perfectionistic strivings Quiz on cognitive distortions Interactive elements where individuals reflect on and share their personal experiences in response to questions
(4) Overcoming fear of mistakes	Psychological education through video content Cognitive restructuring related to the fear of mistakes Practicing objective assessment of mistakes through role play Reevaluating mistakes and recognizing their positive aspects through case studies Interactive elements where individuals reflect on and share their personal experiences in response to questions
(5) Success strategies for perfectionists	Online psychological assessment (post-intervention) Reviewing progress: reinforcing excellencism, reducing perfectionistic strivings. Checking changes and creating future action plans Cognitive restructuring: writing a letter to a friend Comparing changes between Session 1 and Session 5 to assess progress and encourage further self- management

### 2.3 Outcome measures

#### 2.3.1 Frost Multidimensional Perfectionism Scale (FMPS)

The FMPS measures trait perfectionism via 35 items across six dimensions: Concern over Mistakes, Doubts about Actions, Personal Standards, Organization, Parental Expectations, and Parental Criticism. Each item is rated from 1 (strongly disagree) to 5 (strongly agree), and subscale scores can be summed to yield an overall perfectionism score (higher scores indicate greater perfectionism). The FMPS demonstrates high internal consistency (Cronbach's *α* for subscales = 0.77–0.93) and good construct validity (Frost et al., 1990). In this study, the primary outcome measure was the Korean version of the Concern over Mistakes subscale from the FMPS (Lee and Park, 2011), which assesses the cognitive and emotional impact of fearing mistakes. The Concern over Mistakes subscale, which quantifies the tendency to dwell on errors and anticipate negative evaluation from others—a central feature of perfectionism involving harsh self-criticism and interpreting mistakes as personal failures—was selected as the primary outcome because it is theoretically aligned with the core targets of CBT in treating perfectionism (Woodfin et al., 2020) and is the FMPS facet most consistently associated with psychological distress and treatment responsiveness (Larionow, 2024; Rozental et al., 2018; Shafran et al., 2017). The remaining FMPS subscales were included as secondary outcome measures to capture additional dimensions of perfectionism.

#### 2.3.2 Patient Health Questionnaire-9 (PHQ-9)

The PHQ-9 is a 9-item self-report measure of depressive symptom severity based on DSM-IV criteria. Items are rated on a 0–3 scale (0 = not at all; 3 = nearly every day), yielding a total score from 0 to 27. Cutoff scores of 5, 10, 15, and 20 indicate mild to severe depression, with a score ≥ 10 offering 88.0 % sensitivity and specificity for major depression. The PHQ-9 has shown excellent internal reliability (Cronbach's *α* = 0.86–0.89) and validity as a screening and outcome measure (Kroenke et al., 2001). It is also sensitive to clinical change, allowing for effective monitoring of treatment response over time (Lowe et al., 2004).

#### 2.3.3 Generalized Anxiety Disorder-7 (GAD-7)

The GAD-7 is a brief 7-item scale assessing generalized anxiety symptoms severity. Respondents rate how often they have been bothered by each symptom over the last two weeks on a 0–3 scale (0 = not at all; 3 = nearly every day), and the items are summed to yield a total score from 0 to 21, with higher scores indicating greater anxiety. A score of 10 or above is a recommended cutoff indicating possible generalized anxiety disorder. The GAD-7 exhibits excellent internal consistency (Cronbach's *α* = 0.92), good test–retest reliability, and strong criterion validity for identifying GAD cases in clinical and general populations (Spitzer et al., 2006).

#### 2.3.4 Perceived Stress Scale (PSS)

The PSS measures the degree to which situations in one's life are appraised as stressful, emphasizing feelings of unpredictability and lack of control in the past month (Cohen et al., 1983). It includes 10 items rated on a 5-point Likert scale from 0 (never) to 4 (very often), with four positively phrased items reverse-scored. A total perceived stress score (range 0–40) is obtained by summing all items, where higher scores reflect greater perceived stress. The PSS has shown acceptable internal consistency (Cronbach's *α* ≥ 0.70) and good psychometric properties across diverse samples, including stable factor structure and expected correlations with health outcomes and other stress measures (Lee et al., 2015).

#### 2.3.5 Satisfaction With Life Scale (SWLS)

The SWLS (Diener et al., 1985) is a 5-item instrument for global life satisfaction, capturing an individual's conscious evaluative judgment of their life as a whole (Diener et al., 2013). Each item is rated on a 7-point scale from 1 (strongly disagree) to 7 (strongly agree), and the five items are summed to yield a total life satisfaction score ranging from 5 to 35 (higher scores denote greater satisfaction with life). The SWLS has demonstrated high internal reliability (Cronbach's *α* = 0.87).

### 2.4 Statistical analysis

Statistical analyses were conducted using R software, version 4.2.3. All statistical analyses adhered to the intention-to-treat principle, utilizing mixed-effects models to evaluate the intervention's efficacy on both primary and secondary outcome measures. These models included fixed effects for time, group, and their interaction to account for changes over the course of the intervention. To address missing data in the post-intervention assessments, a multiple imputation strategy was applied using the ‘mice’ package in R. Predictive mean matching (PMM) was employed to manage missingness effectively, generating 20 imputed datasets to enhance the robustness of the analyses. The predictor matrix included baseline variables (group assignment, age, sex, and pre-treatment outcome scores), and self-prediction was excluded by zeroing the diagonal. Missingness in post-treatment FMPS Concern over Mistakes scores occurred for 41.2 % of the ICBT group and 34.0 % of the control group; however, this difference was not statistically significant (*χ*^*2*^ = 0.29, *p* = .59). Results from the imputed datasets were pooled using Rubin's rules. Between-group effect sizes were calculated as Cohen's *d*, based on mean differences between conditions and normalized by the pooled standard deviation, with 95 % confidence intervals (CIs) reported.

The Reliable Change Index (RCI) was used to identify clinically meaningful changes in outcome measures. An observed change exceeding the RCI threshold was interpreted as a significant clinical improvement. The RCI was calculated as RCI = SE_diff_ × 1.96, where SE_diff_ = SD1√2√(1-r) (Evans et al., 1998; Jacobson and Truax, 1991). This approach ensured that improvements were not only statistically significant but also clinically relevant. In addition, the percentage change from pre- to post-treatment was calculated for each participant using the formula [(Pre − Post) / Pre] × 100, providing a complementary indicator of clinical improvement. Both RCI and percentage change analyses were conducted only for participants who completed the post-intervention assessments, as these metrics require complete pre- and post-treatment data.

Additionally, a multiple linear regression analysis was conducted to identify predictors of treatment outcomes, defined as changes in FMPS Concern over Mistakes scores. Predictor variables included baseline demographic and clinical characteristics. Results were reported as standardized beta coefficients (β) with 95 % CIs, and statistical significance was defined as *p* < .05. Internal consistency (Cronbach's α) coefficients were calculated for each outcome measure using pre-treatment data to assess the reliability of the instruments.

## 3 Results

### 3.1 Baseline characteristics, attrition, and adherence

At baseline, no significant between-group differences were found for any clinical outcome measure except perceived stress. Specifically, the waitlist group reported significantly higher perceived stress scores (PSS: 24.50 ± 6.19) compared to the ICBT group (PSS: 21.72 ± 3.51; *t* = −2.65, *p* = .010).

Attrition rates were 41.2 % in the ICBT group (*n* = 21) and 36.0 % in the waitlist control group (*n* = 18). Comparisons between participants who completed the post-intervention assessment and those who did not revealed no significant differences in demographic or baseline clinical characteristics. The average age of completers was 31.55 ± 7.83 years, compared to 30.44 ± 6.84 years for non-completers (*t* = 0.752, *p* = .454). Gender distribution was similar, with 72.6 % of completers being female and 27.4 % male, versus 69.2 % female and 30.8 % male among non- completers (*χ*^*2*^ = 0.019, *p* = .892). Baseline scores on the FMPS Concern over Mistakes were also comparable, with completers scoring an average of 32.62 ± 5.11 and non-completers 33.62 ± 4.64 (*t* = 1.016, *p* = .312). In the ICBT group, participants accessed an average of 3.71 out of 5 available modules (SD = 1.71), with 58.8 % fully completing all five modules (Table 2).

**Table 2:** Number of opened modules during treatment.

	ICBT group (*n* = 51)
One module	49 (96.1 %)
Two modules	43 (84.3 %)
Three modules	35 (68.6 %)
Four modules	34 (66.7 %)
Five modules	30 (58.8 %)

ICBT, internet-based cognitive behavioral therapy.

### 3.2 Treatment results

Descriptive statistics for the primary and secondary outcome measures across the respective conditions are presented in Table 3 and Supplementary Fig. 1. Table 3 also presents Cronbach's *α* coefficients for each outcome measure and FMPS subscale, all of which indicated acceptable to excellent internal consistency (*α* = 0.72–0.90), supporting the reliability of the scales used in this study. Between-group effect sizes and their 95 % CIs are shown in Table 4. For the primary outcome measure, FMPS Concern over Mistakes, the ICBT group demonstrated a significant reduction in scores, from a mean of 32.63 at baseline to 28.25 post-intervention. This reduction was statistically significant, with a time*group interaction (*β* = −3.91, *p* < .001) and a large between-group effect size (Cohen's *d* = −0.65, 95 % CI: [−1.05, −0.25]). For the FMPS Organization subscale, a significant time*group interaction was observed (*β* = −1.44, *p* = .014); however, the between-group effect size was relatively small, and the CI included zero (*d* = −0.07, 95 % CI: [−0.47, 0.34]). Other FMPS subscales, including Personal Standards, Parental Expectations, Parental Criticism, and Doubts about Action, showed reductions in scores, but these changes were not statistically significant for time*group interactions or between-group effect sizes.

Among secondary outcomes, PHQ-9 scores significantly decreased, reflecting improvement in depressive symptoms, with a time*group interaction (*β* = −2.47, *p* = .028). However, the CI for the between-group effect size included zero, suggesting limited evidence for a clinically meaningful difference. PSS scores significantly decreased (*β* = 2.08, *p* = .004), though the effect size indicated no significant difference between groups. GAD-7 scores significantly decreased (*β* = −3.04, *p* = .001), with a moderate between-group effect size (*d* = −0.42, 95 % CI: [−0.83, −0.01]), demonstrating the intervention's efficacy in reducing anxiety symptoms. Finally, SWLS scores significantly increased, indicating improved life satisfaction (*β* = 1.78, *p* = .018), with a moderate and significant effect size (*d* = 0.62, 95 % CI: [0.20, 1.03]).

**Table 3:** Reliability coefficients (Cronbach's *α*), means (standard deviations), and sample sizes of outcome variables across groups over time.

Outcomes	Cronbach's *α*	ICBT			Waitlist		
		Pre	Post	n	Pre	Post	n
FMPS	0.90						
Concerns over mistakes	0.72	32.63 (5.25)	28.25 (7.74)	51	33.38 (4.62)	32.91 (6.58)	50
Personal standards	0.78	24.54 (5.66)	22.26 (6.31)	48	25.54 (4.64)	24.35 (5.29)	46
Parental expectations	0.89	12.94 (5.05)	12.13 (5.21)	48	13.24 (6.36)	12.92 (5.99)	46
Parental criticism	0.86	9.73 (4.07)	8.83 (3.96)	48	10.76 (4.72)	10.20 (4.41)	46
Doubts about action	0.74	13.75 (3.48)	12.66 (4.02)	48	14.02 (3.53)	13.62 (3.90)	46
Organization	0.88	20.33 (6.16)	18.14 (6.03)	48	19.28 (5.27)	18.53 (5.27)	46
PHQ-9	0.87	12.52 (7.02)	10.41 (7.41)	48	12.28 (6.69)	12.64 (6.84)	46
GAD-7	0.87	10.79 (5.68)	8.49 (6.65)	47	10.43 (5.05)	11.18 (6.18)	46
PSS	0.77	21.72 (3.51)	20.64 (3.65)	47	24.50 (6.19)	21.35 (3.36)	46
SWLS	0.85	16.51 (5.59)	18.78 (6.17)	47	14.48 (6.37)	14.97 (6.23)	46

ICBT, internet-based cognitive behavioral therapy; FMPS, Frost Multidimensional Perfectionism Scale; PHQ-9, Patient Health Questionnaire-9; GAD-7, Generalized Anxiety Disorder-7; PSS, Perceived Stress Scale; SWLS, Satisfaction With Life Scale.

**Table 4:** Between-group effect sizes and Cohen *d* [95 % CI] for all outcome measures.

	LMM estimate		Between-group effect size
	*β*^a^	*p*	Cohen's *d* [95 % CI]
FMPS			
Concerns over mistakes	−3.91	.001	−0.65 [−1.05, −0.25]
Personal standards	−1.09	.128	−0.36 [−0.77, 0.05]
Parental expectations	−0.49	.307	−0.14 [−0.55, 0.26]
Parental criticism	−0.34	.520	−0.33 [−0.74, 0.08]
Doubts about action	−0.69	.180	−0.24 [−0.65, 0.16]
Organization	−1.44	.014	−0.07 [−0.47, 0.34]
PHQ-9	−2.47	.028	−0.31 [−0.72, 0.09]
GAD-7	−3.04	.001	−0.42 [−0.83, −0.01]
PSS	2.08	.004	−0.20 [−0.61, 0.21]
SWLS	1.78	.018	0.62 [0.20, 1.03]

CI, confidence interval; FMPS, Frost Multidimensional Perfectionism Scale; PHQ-9, Patient Health Questionnaire-9; GAD-7, Generalized Anxiety Disorder-7; PSS, Perceived Stress Scale; SWLS, Satisfaction With Life Scale.

a *β* = time ∗ group interaction.

### 3.3 Improvement and deterioration

In the ICBT group, among participants who completed the post-intervention assessment, scores on the FMPS Concern over Mistakes decreased for 22 individuals, increased for 7 individuals, and remained unchanged for one. The RCI, calculated at 10.07 based on a Cronbach's alpha of 0.72 for this study, was employed to determine clinically meaningful changes. Among those with decreased scores, 11 participants (representing 36.7 % of those completing the post-intervention assessment in the ICBT group) exhibited reductions that exceeded the RCI threshold, indicating significant clinical improvement. Consistent with this, the average percentage reduction in FMPS Concern over Mistakes scores among ICBT participants was 19.7 % (SD = 24.33), while the waitlist group showed a mean increase of 3.4 % (SD = 16.46). Conversely, none of the participants with increased scores surpassed the RCI value, suggesting that the intervention specifically facilitated clinically meaningful reductions in perfectionism concerns without inducing worsening in a clinically significant manner.

### 3.4 Predictors of treatment outcome

The multiple regression analysis identified baseline FMPS Concern over Mistakes scores as a significant predictor of post-intervention FMPS Concern over Mistakes score reductions (*β* = −0.71, 95 % CI: [−1.60, −0.61]), indicating that individuals with higher baseline levels of perfectionism experienced greater improvements. Demographic characteristics (age, gender, marital status, education level, and employment status), number of modules completed, and other baseline clinical measures (PHQ-9, GAD-7, PSS, SWLS, and FMPS subscales) did not significantly predict treatment outcomes (Table 5).

**Table 5:** Multiple linear regression analysis of predictors of treatment outcome, using changes in FMPS Concern over Mistakes scores as the dependent variable.

Predictor variables	*b*	*β*	SE	95 % CI	
				Lower	Upper
Age	−0.15	−0.16	0.17	−0.49	0.18
Gender (male)	−0.19	−1.46	2.22	−5.80	2.89
Marital status (single)	−0.07	−0.54	3.11	−6.63	5.55
Education level (university)	−0.04	−0.34	2.63	−5.50	4.83
Education level (graduate school)	0.17	1.32	3.81	−6.15	8.80
Employment	−0.14	−1.05	2.23	−5.42	3.32
Baseline FMPS scores					
Concern over mistakes	−0.71	−1.11	0.25	−1.60	−0.61
Personal standards	0.25	0.38	0.24	−0.10	0.85
Parental expectations	0.05	0.06	0.27	−0.47	0.60
Parental criticism	0.12	0.22	0.39	−0.55	0.98
Doubts about action	0.00	−0.01	0.35	−0.70	0.68
Organization	0.07	0.09	0.21	−0.31	0.50
Baseline PHQ-9 score	0.02	0.02	0.23	−0.42	0.46
Baseline GAD-7 score	0.09	0.13	0.31	−0.48	0.74
Baseline PSS score	0.21	0.31	0.26	−0.20	0.82
Baseline SWLS score	−0.14	−0.18	0.19	−0.54	0.19
Number of completed modules	−0.17	−0.62	0.53	−1.64	0.41

FMPS, Frost Multidimensional Perfectionism Scale; PHQ-9, Patient Health Questionnaire-9; GAD-7, Generalized Anxiety Disorder-7; PSS, Perceived Stress Scale; SWLS, Satisfaction With Life Scale.

## 4 Discussion

The current study is the first RCT in South Korea to evaluate the efficacy of unguided ICBT for individuals with elevated levels of perfectionism, specifically targeting rigid and self-critical perfectionistic strivings through five structured modules. Significant improvements were found in the primary outcome measure, the FMPS Concern over Mistakes, with the ICBT group showing greater reductions in perfectionism symptoms compared to the waitlist control group. Furthermore, significant improvements were observed in secondary outcomes such as depression, anxiety, and life satisfaction, highlighting the broader therapeutic potential of ICBT. These findings suggest that ICBT could serve as an effective and scalable intervention for addressing perfectionistic strivings and associated psychological distress across diverse populations.

As hypothesized, our results demonstrate that the treatment group exhibited significantly greater reductions in scores on the FMPS Concern over Mistakes subscale compared to the waitlist control group, with a between-group effect size of −0.65. These findings are consistent with previous studies (Grieve et al., 2022; Rozental et al., 2017; Wade et al., 2019), such as Rozental et al. (2017), which also reported moderate to large effect sizes for ICBT in addressing perfectionism-related behaviors. Notably, despite using an unguided ICBT, our effect size was comparable to that of Rozental et al. (2017), which employed guided ICBT. This suggests that even without therapist support, our ICBT program can effectively reduce perfectionistic strivings, underscoring its scalability and potential as a cost-effective, low-resource intervention. Additionally, a recent meta-analysis reported that face-to-face CBT for perfectionism yielded moderate to large effect sizes in domains such as Personal Standards and Concern over Mistakes (Galloway et al., 2022). The effect sizes in our study for ICBT were comparable, indicating that ICBT is similarly effective in reducing perfectionistic strivings. While future comparative effectiveness studies between ICBT and face-to-face CBT are warranted, the current findings suggest that ICBT is a viable alternative to conventional therapy, particularly for individuals facing barriers to in-person treatment.

In addition to the primary outcome, the ICBT intervention showed significant improvements in secondary outcomes, including reductions in anxiety and life satisfaction, supporting its broad therapeutic benefits. These findings align with prior research, where guided ICBT for perfectionism demonstrated moderate effect sizes for depression and anxiety and large effect sizes for quality of life (Rozental et al., 2017). Similarly, Shu et al. (2019) reported that ICBT reduced symptoms of depression, anxiety, and eating disorders, while improving self-esteem, with effects maintained at 6-month follow-up. In contrast, Grieve et al. (2022) found no significant between-group differences in secondary outcomes for unguided ICBT. However, our results indicate that even unguided ICBT can lead to significant improvements in these symptoms. This suggests that both guided and unguided ICBT for perfectionism may have a transdiagnostic impact (Egan et al., 2011), alleviating various psychological conditions by targeting perfectionistic strivings, which is closely linked to several psychiatric disorders (Egan et al., 2014; Handley et al., 2015; Rozental et al., 2017; Shu et al., 2019).

We did not observe significant changes in other perfectionism subscales, such as Doubts about Actions, Parental Expectations, and Parental Criticism. This may relate to the nature and focus of our intervention. These subscales represent more deeply ingrained and externally rooted dimensions of perfectionism, often reflecting internalized standards shaped by earlier parenting styles, and therefore less responsive to short-term therapeutic interventions (Damian et al., 2013; Zikopoulou et al., 2021). Indeed, parental-related subscales are considered developmental precursors rather than core drivers of present psychological distress, often showing minimal change during treatment (Curran and Hill, 2019; Flett et al., 2002). Furthermore, perfectionism exhibits partly trait-like stability (Stöber, 1998), meaning individuals tend to maintain stable relative levels despite mean-level improvements (Maia et al., 2011), suggesting that more intensive or extended interventions might be required to modify chronic doubts or deeply internalized parental expectations.

In this study, 36.7 % of participants in the ICBT group who completed post-intervention assessments demonstrated clinically significant improvements in perfectionistic strivings, as indicated by exceeding the RCI threshold. Comparatively, Arana et al. (2017) reported that 75 % of participants in a face-to-face CBT program involving five two-hour sessions per week achieved reliable change. The relatively lower rate of improvement in our study may be attributed to the unguided nature of the ICBT intervention, which lacked the direct therapist support provided in traditional CBT format. Indeed, a clinical trial by Egan et al. (2014) comparing face-to-face CBT with online self-help CBT for perfectionism reported that 67 % of participants in the face-to-face group and 40 % in the unguided group met the criteria for recovery at follow- up. This implies that while therapist involvement may enhance treatment outcomes, unguided interventions can still yield meaningful improvements. Furthermore, none of the participants with increased FMPS Concern over Mistakes scores exceeded the RCI threshold, suggesting that the observed deterioration was not clinically meaningful. These findings indicate the effectiveness of the ICBT program in reducing perfectionistic strivings, with no indication of true clinical worsening despite minor score increases in some participants.

Our multiple regression analysis showed that participants with higher initial levels of perfectionism experienced greater reductions in these concerns following the ICBT program. This finding aligns with previous research suggesting that baseline severity predicts treatment response in cognitive-behavioral interventions (Scholten et al., 2023). These results indicate that ICBT may be particularly beneficial for individuals with pronounced perfectionistic tendencies. This association between baseline severity and treatment outcomes has practical implications for future interventions. Screening measures to identify individuals with higher perfectionism could enable tailored intervention delivery, such as prioritizing intensive or guided support for those most likely to benefit. Stratified approaches or targeted recruitment of individuals with severe perfectionism might further enhance the efficacy and efficiency of ICBT programs. Interestingly, demographic variables and other baseline clinical measures did not significantly predict treatment outcomes. This is consistent with prior ICBT studies reporting that demographic and clinical factors, selected a priori, were not significantly associated with intervention efficacy (Rozental et al., 2017). Rather, this lack of association suggests that ICBT programs for perfectionism may be broadly applicable across diverse demographic and clinical profiles.

### 4.1 Limitations

This study has several limitations that should be considered when interpreting the findings. First, the attrition rate in the ICBT group was relatively high at 41.2 %, though comparable to previous ICBT research on perfectionism (Grieve et al., 2022; Rozental et al., 2018). However, it remains higher than the attrition rates reported in face-to-face CBT (16.7 %) and some guided ICBT studies (12.2–14.1 %) (Arana et al., 2017; Rozental et al., 2017). This may limit the generalizability of our results. The unguided nature of the ICBT program, while promoting accessibility, may have contributed to the dropout rate. Future studies could explore methods to enhance participant engagement and retention, such as incorporating minimal therapist support or automated reminders. Second, this study only included pre- and post-treatment assessments, with no follow-up evaluations, and the timing of post-assessment in the ICBT group varied depending on whether participants completed all modules. This limits our ability to determine whether the observed improvements in perfectionistic strivings and related symptoms are maintained over time or influenced by differences in the timing of assessments (Rozental et al., 2018; Shu et al., 2019). Future studies should incorporate standardized follow-up assessments (e.g., at 3- or 6-month post-intervention) to evaluate the sustainability of treatment effects, particularly important given the unguided format of the intervention. Third, only some participants in the ICBT group showed clinically significant improvement. However, we were unable to identify predictors of treatment outcome that could explain why certain participants responded to the intervention while others did not. This leaves uncertainty regarding which individuals are most likely to benefit from the treatment. Fourth, our use of a waitlist control group limits the ability to clearly isolate intervention-specific effects from non-specific factors such as expectancy or the mere passage of time. While waitlist controls are suitable for preliminary studies, future research should consider including more robust comparison conditions, such as active or minimal-intervention controls, to enhance the interpretability of results and reduce potential confounds (Goldberg et al., 2023). Additionally, participant satisfaction and perceived utility of the intervention were not assessed, which limits our understanding of the user experience. Lastly, the broad inclusion and exclusion criteria may have led to a heterogeneous sample, including participation referred from psychiatric clinics as well as those from non-clinic sources. Due to the small and heterogeneous nature of the non-clinic subgroup, referral source was not included as a predictor, as it was unlikely to yield valid or interpretable results. While this variability could have influenced outcomes due to underlying psychiatric comorbidities (Hilvert-Bruce et al., 2012; Karyotaki et al., 2017), it also allowed us to test the ICBT program across a broader spectrum of individuals, enhancing its ecological validity compared to previous studies (Grieve et al., 2022; Rozental et al., 2017, Rozental et al., 2018) that primarily focused on non-clinical populations. Future research could further explore this heterogeneity through subgroup analyses to better account for comorbidities and refine the intervention's applicability.

## 5 Conclusions

Our unguided ICBT intervention demonstrated effectiveness in reducing perfectionistic strivings and associated psychological distress among individuals with elevated levels of perfectionism. If these results are replicated, integrating this intervention into mental health care settings could be a valuable approach to bridging the current treatment gap. ICBT can be effectively incorporated into stepped-care models or self-referral systems, provided adequate digital infrastructure is established. Nevertheless, further research is warranted to confirm its long-term efficacy and to investigate other factors that may predict treatment outcomes. Future studies should also explore innovative strategies to enhance adherence, such as analyzing users' patterns of thought and emotion, and utilizing artificial intelligence (AI) for personalized feedback and guidance. This approach could improve treatment outcomes, engagement, and retention rates.

The following are the supplementary data related to this article.

Supplementary Fig. 1 Changes of subscales of FMPS and other outcome variables over time by group. Pre-, pre-treatment assessment, post, post-treatment assessment; FMPS_CM, Frost Multidimensional Perfectionism Scale-Concern over Mistakes; FMPS_PS, Frost Multidimensional Perfectionism Scale-Personal Standards; FMPS_PE, Frost Multidimensional Perfectionism Scale-Parental Expectations; FMPS_PC, Frost Multidimensional Perfectionism Scale-Doubts about Action; FMPS_OR, Frost Multidimensional Perfectionism Scale-Organization; PHQ_9, Patient Health Questionnaire-9; GAD-7, Generalized Anxiety Disorder-7; PSS, Perceived Stress Scale; SWLS, Satisfaction With Life Scale. Supplementary Fig. 1

## CRediT authorship contribution statement

JL and DUY led the conceptualization and design of the study and coordinated the overall research project. DUY developed the ICBT program used in the study and provided expert insights into its implementation. JL, SO, DUY, YL, and HJL collaboratively recruited participants for the study. JC, JJ, and JHL collected and managed the data. JL and SO performed statistical analysis and contributed significantly to drafting the manuscript. All authors reviewed and approved the final manuscript and agreed to be accountable for all aspects of the work.

## Declaration of competing interest

None declared.
