# Supplementary material for: Efficacy of Internet-based cognitive behavioral therapy for reducing perfectionistic strivings in the Republic of Korea: A randomized controlled trial
Source: Internet Interv. 2025 Jun 29;41:100851. doi: 10.1016/j.invent.2025.100851 (PMC12271590; doi:10.1016/j.invent.2025.100851)
Supplement: Supplementary Fig. 1 — Changes of subscales of FMPS and other outcome variables over time by group. Pre-, pre-treatment assessment, post, post-treatment assessment; FMPS_CM, Frost Multidimensional Perfectionism Scale-Concern over Mistakes; FMPS_PS, Frost Multidimensional Perfectionism Scale-Personal Standards; FMPS_PE, Frost Multidimensional Perfectionism Scale-Parental Expectations; FMPS_PC, Frost Multidimensional Perfectionism Scale-Doubts about Action; FMPS_OR, Frost Multidimensional Perfectionism Scale-Organization; PHQ_9, Patient Health Questionnaire-9; GAD-7, Generalized Anxiety Disorder-7; PSS, Perceived Stress Scale; SWLS, Satisfaction With Life Scale. [file mmc1.docx]

Supplementary Figure 1. Changes of subscales of FMPS and other outcome variables.


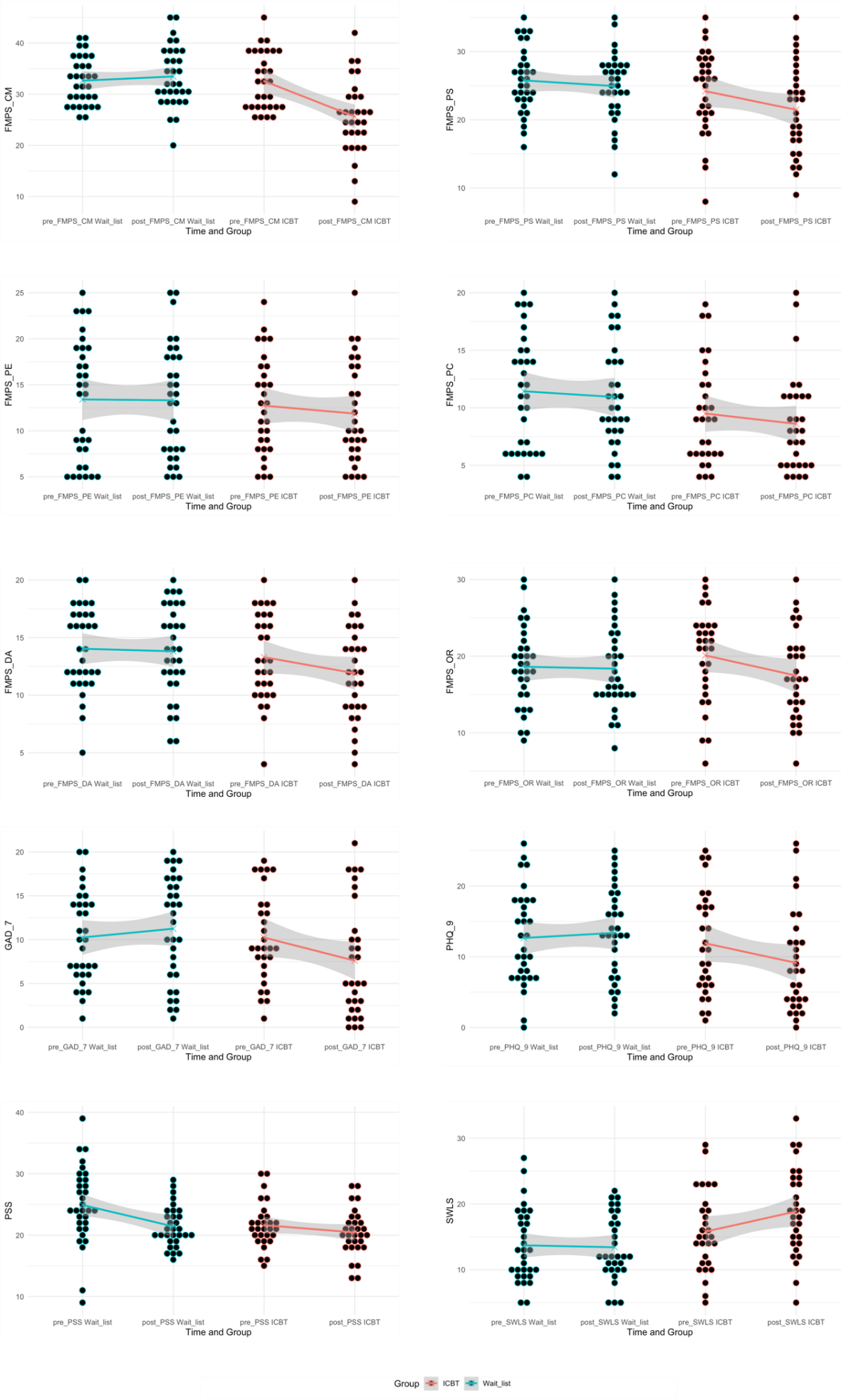


**Supplementary Figure 1.** Changes of subscales of FMPS and other outcome variables over time by group. pre-, pre-treatment assessment, post, post-treatment assessment; FMPS_CM, Frost Multidimensional Perfectionism Scale-Concern over Mistakes; FMPS_PS, Frost Multidimensional Perfectionism Scale-Personal Standards; FMPS_PE, Frost Multidimensional Perfectionism Scale-Parental Expectations; FMPS_PC, Frost Multidimensional Perfectionism Scale-Doubts about Action; FMPS_OR, Frost Multidimensional Perfectionism Scale-Organization; PHQ_9, Patient Health Questionnaire-9; GAD-7, Generalized Anxiety Disorder-7; PSS, Perceived Stress Scale; SWLS, Satisfaction With Life Scale.
